# 13-year-old Girl with Fevers

**DOI:** 10.5811/cpcem.2017.3.34163

**Published:** 2017-03-21

**Authors:** Megan Cobb, Emily Gorman, Laura J Bontempo, Zachary DW Dezman

**Affiliations:** * University of Maryland Medical Center, Baltimore, Maryland; † University of Maryland School of Medicine, Department of Emergency Medicine, Baltimore, Maryland

## CASE PRESENTATION

A 13-year-old African-American female presented to our pediatric emergency department (ED) with fever for eight days, as high as 103°F at home. Her fevers responded to antipyretics but would return without an identifiable pattern, often within a few hours, sometimes longer; but the fever would always return by the next day. She also complained of odynophagia, headache, fatigue, and dizziness. The patient had been seen in another ED two days prior for these symptoms. She had been diagnosed as having streptococcal pharyngitis because of a positive rapid test and was prescribed amoxicillin. She came into our ED because her symptoms had worsened despite compliance with her antibiotics. Specifically, the patient had developed gradually worsening joint pains in her ankles, knees and shoulders. Her mother noted the patient’s joints had become swollen that day and she had developed a facial rash. The patient denied any nausea, vomiting, diarrhea, abdominal pain, or respiratory symptoms such as coughing or wheezing.

Her past medical history included seizures of an unknown etiology occurring between birth and three years of age. She saw her pediatrician regularly. While she lacked a current influenza vaccination, all of her other immunizations were up to date. Her only medication was the amoxicillin she had been prescribed two days prior to presentation. She had no known drug allergies. Her family medical history was significant for a sibling with idiopathic pancreatitis and several family members with type 2 diabetes and hypertension. The patient had not travelled recently, she was post-menarchal, and was not sexually active. Her last menstrual period was two weeks prior to presentation. When questioned without family present, the patient denied any form of abuse.

At the time of her ED evaluation, the patient was afebrile (37.2°C) with a heart rate of 80 beats/minute, respiratory rate of 20 breaths/minute, blood pressure of 118/73 mmHg, and she had an oxygen saturation of 99% on room air. She was 5 feet 1 inch tall (1.35m) and weighed 90 pounds (41kg), giving her a body mass index of 17. This placed her at the 25th percentile for height and weight for her age.

Physical examination revealed a well-developed and nourished adolescent female in no acute distress who appeared fatigued. Her head was normocephalic and atraumatic with bilateral periorbital edema. Her conjunctiva and sclera were normal. Her mucous membranes were moist and she had no nasal discharge. The posterior oropharynx was erythematous without exudates. Her tongue was normal and no intra-oral lesions were seen. Her neck was supple with bilateral cervical lymphadenopathy, the largest node measuring 1.5cm in length. Her lungs were clear to auscultation bilaterally. Her heart was regular without murmurs, rubs or gallop. Her abdomen was soft and non-tender without organomegaly.

She had mild joint pain with passive movement of her extremities, but she had full range of motion except for pain-limited plantar and dorsiflexion of her ankles. There was bilateral pedal edema. Examination of her skin revealed an erythematous midface rash with nasolabial and perioral sparing. No rashes were present elsewhere. Her cranial nerves were intact. The patient had full motor strength throughout all of her extremities. Her coordination, balance, speech, and comprehension were all normal.

Her initial laboratory results are shown in Tables 1 and 2. Based on the suspicion of the clinician, an additional laboratory test was sent that confirmed the diagnosis.

## CASE DISCUSSION

What immediately struck me about this case was that this 13-year-old girl was sick. A fever for eight days, not getting better and having an increasing number of symptoms despite taking amoxicillin for a presumed strep throat raised my suspicions that something bad was going on. My initial thoughts were infectious versus autoimmune etiologies.

This case contained so much information that the first challenge was to focus on the pertinent features. The patient’s fever, rash, joint pain, periorbital edema, pedal edema, and thrombocytopenia seemed to all be important clues. The patient’s laboratory work also suggested a hemolytic anemia (low hematocrit with elevated lactate dehydrogenase [LDH]). I did not believe amoxicillin played a role in the patient’s presentation, and in fact it could have been a red herring. The secondary clues that I thought were important for narrowing down the differential included the fatigue, headache, sore throat, lack of a flu shot, normal renal function and a sibling with pancreatitis. I split my initial differential diagnoses into the same three categories I use every day in the ED: life-threats; common; and rare.

- **Life-Threats**
  Meningitis, herpes simplex virus (HSV) encephalitis, Kawasaki disease, pericarditis, endocarditis, hemolytic-uremic syndrome (HUS), thrombotic thrombocytopenic purpura (TTP), pancreatitis
- **Common**
  Influenza, juvenile rheumatoid arthritis (JRA), mononucleosis, drug rash/fever, mycoplasma
- **Rare**
  Measles, rheumatic fever (RF), disseminated gonococcus, malaria, systemic lupus erythematosus (SLE), Zika virus, scarlet fever, tertiary syphilis

There is a large differential for fever, and we can’t easily test for all of these diagnoses in the ED. But this challenge is the crux of emergency medicine – being able to think of all of these possibilities and work through why each is or isn’t a possible cause of the patient’s symptoms. There were several I was able to cross off the list quickly.

- **Meningitis/Encephalitis**
  I was able to cross these off the list because the patient had a supple neck, a normal neurological exam, no known exposure to HSV, and while the patient had a rash, it was inconsistent with meningococcus.
- **Kawasaki Disease**
  The patient was older than the usual age range (under five years old), and she did not have the conjunctivitis, a “strawberry tongue,” or rash consistent with Kawasaki disease.
- **Pericarditis/Endocarditis**
  The patient did not have a precordial rub, murmur, or chest pain. As it wasn’t a part of the presented history, I am assuming the patient did not use intravenous street drugs and therefore was not at elevated risk for endocarditis.
- **Drug Rash/Fever**
  The patient’s symptoms had been present for nearly a week prior to her being given amoxicillin. The patient did not take any other medications.
- **Mycoplasma**
  The lack of cough or other respiratory symptoms make this diagnosis less likely.
- **Measles**
  The patient was vaccinated, did not have any sick contacts or exposures to measles, and was not known to be immunocompromised.
- **Disseminated Gonococcus/Syphilis**
  While we should always be skeptical in emergency medicine, the patient stated she was not sexually active and had no history of congenital infections.
- **Malaria/Zika**
  The patient had not travelled recently and had no history of contact with a known case of either disease.
- **Scarlet Fever**
  The patient had a positive rapid strep test, but she didn’t have the all-over sandpaper rash classically associated with scarlet fever. Similarly, hemolytic anemia is not known to be associated with scarlet fever.

Once I had narrowed my differential to the eight remaining diagnoses, I went through each one, listing history and physical exam items that pointed to or away from each possibility.

### HUS-TTP spectrum

Crude as it is, I remembered the “FAT RN” mnemonic for TTP – **F**ever, **A**nemia, **T**hrombocytopenia, **R**enal failure, **N**eurologic symptoms. This patient had a fever, anemia, and thrombocytopenia. A urinalysis, especially if it showed proteinuria, would have been helpful. The patient’s creatinine was normal, however, which is not consistent with this diagnosis. Headache may be considered a neurologic symptom, but that is a soft call. HUS is usually characterized by diarrhea, which our patient did not have. The rash was only on her face and not the typical purpura of TTP. Based on this, I felt HUS-TTP spectrum was unlikely to be the diagnosis and crossed it off my differential.

### Pancreatitis

Pancreatitis can have a rare complication of hemolytic anemia, which would support this diagnosis. The patient’s LDH was elevated, and her sibling presumably had hereditary pancreatitis. I was missing the lipase, but in light of the fact that the patient did not have abdominal pain or vomiting and had so many other symptoms that did not fit with pancreatitis, I felt that pancreatitis was unlikely and crossed it off my differential.

### Influenza

The patient had not been vaccinated, so influenza was still on the differential. Fever, joint aches, headache and fatigue all pointed toward a flu-like illness. However, hemolytic anemia is a very rare complication and influenza has not been known to cause pedal edema or a facial rash. I therefore crossed influenza off the list.

### Rheumatic Fever

I personally have never seen this rare^1^ disease. However, the patient’s fever, joint pain and positive rapid strep test made me seriously consider rheumatic fever. The patient did not have a murmur, chorea, or an all-over rash, all of which pointed against RF. Thrombocytopenia is not a known complication of the disease. Given all the clues that point away from this diagnosis and the rarity of it, I crossed rheumatic fever off the list.

### Mononucleosis

The patient had many historical and physical exam clues that pointed towards this diagnosis. She was the right age and she had a fever, sore throat, fatigue, and lymphadenopathy. The development of a rash after taking amoxicillin for presumed streptococcal pharyngitis, when the true cause is mononucleosis, is a well-documented phenomenon.^2^ While it is rare, hemolytic anemia is a complication of mononucleosis. The patient’s facial rash points away from mononucleosis, however, since that rash is typically widespread. Pedal edema and joint pain are also not typical of mononucleosis. The patient did not have splenomegaly, although this can be difficult to detect on exam, or it might not yet have developed at this point in the disease. In the end, the heterophile antibody test can be run quickly and easily in the ED, so I kept mononucleosis on my differential.

### JRA/SLE

I have a hard time separating these two diseases. SLE has a malar rash and is associated with constitutional symptoms like those in this case, so I focused on SLE. It is rare but SLE can present in the teenage years. Our patient had many of the classic symptoms – malar rash, joint pain, edema, hemolytic anemia, and thrombocytopenia, as well as decreased dorsi- and plantar-flexion. Although she did not have renal involvement, this is often a later development of the disease and does not necessarily point away from the diagnosis. According to the Systemic Lupus International Collaborating Clinics/American College of Rheumatology, four or more of the following criteria are needed to make the diagnosis of SLE: cutaneous involvement, renal impairment, neurologic symptoms, hematologic involvement, arthritic symptoms, serositis, and positive immunological tests.^3^ Our patient had a malar rash (cutaneous), hemolytic anemia and thrombocytopenia (hematologic), joint pain and edema (arthritic). If she had either a positive antinuclear antibody (ANA) or anti-double-stranded DNA (anti-dsDNA) test, she would have SLE. SLE therefore remained on my differential diagnosis list.

My final differential included mononucleosis or SLE. In the real world I would send tests looking for heterophile, antinuclear and anti-dsDNA antibodies, and admit the patient for further workup/management. In the winter months, I would also send a flu swab. But the malar rash is classic for SLE and not for mononucleosis or influenza. Serum ANA can be elevated in several autoimmune syndromes;^4^ so, if I were forced to choose one diagnosis and one test, I would pick SLE and test for anti-dsDNA antibodies.

## CASE OUTCOME

The diagnostic test sent was an anti-dsDNA antibody. Her level was significantly elevated at 97 IU/mL (normal range <5 IU/mL). She was formally diagnosed with SLE and started on high-dose glucocorticoids. Her joint pain and swelling responded well to intravenous methylprednisolone over the following three days. On hospital day four, she was transitioned to a 20-day prednisone taper. She was given ranitidine to prevent steroid-induced gastrointestinal distress and acetaminophen for pain, and was discharged home.

Two months later the patient was transitioned from daily prednisone to hydroxychloroquine. She experienced a near-complete resolution of the physical signs of SLE but continued to complain of fatigue two to three times per week and occasional joint pains. Her hemoglobin improved from 8.7 g/dL to 11.4 g/dL. Her sedimentation rate decreased from 125 mm/hour to 72 mm/hour (normal 0–29 mm/hr for women), and her c-reactive protein decreased from 2.2 mg/L to 0.9 mg/L (normal <0.8 mg/L).

## RESIDENT DISCUSSION

SLE is found in all age groups – 1–6:100,000 in children and 1:1,500 in adults.^5^,^6^ The incidence increases to 1:700 in women of childbearing age.^6^,^7^ Juvenile SLE is often diagnosed between prepubescent and early adolescent ages. The disease is nine times more common in women, and two to three times more common in African Americans and Latinos than Caucasians.^6^,^8^ Because SLE is so rare in the pediatric population, patients often undergo multiple negative workups before they are correctly diagnosed.^9^,^10^ The prolonged fever, rash and swelling may be mistaken for Kawasaki disease or streptococcal infections. Arthralgias may be called growing pains. The fatigue, malaise and nonspecific symptoms may be attributed to a viral syndrome. Only when taken in total do these often non-specific symptoms point toward SLE.

The pathophysiology begins with the formation of apoptotic cellular bodies and an incomplete clearance of intracellular debris. The release of auto-antigens stimulates immune complex formation with auto-antibodies. Most commonly these are anti-nuclear, anti-dsDNA, anti-Smith, and anti-histone autoantibodies.^10^ Once the immune complexes form, they deposit into tissues throughout the body, causing a systemic vasculitis through a type-III hypersensitivity reaction. Though rare in children, 40–50% of adult patients with SLE also produce an anti-phospholipid autoantibody, which inactivates prothrombin, protein C, and protein S.^11^ These are critical regulatory enzymes in the coagulation cascade, and their inactivation is responsible for the increased risk of bleeding and thromboembolic events found in patients with SLE.

Based on the 2015 guidelines by the American College of Rheumatology and Systemic Lupus International Collaborating Clinics, the revised diagnostic criteria for SLE include cutaneous and neuropsychiatric manifestations, renal and hematologic abnormalities, and autoimmune laboratory values.^3^,^12^ In a multicenter study, childhood-onset SLE symptoms were most commonly hematologic (72%), cutaneous (70%), musculoskeletal (64%), renal (50%), and fever (58%).^10^ It is unlikely that a patient will display enough findings during one ED visit for a definitive diagnosis.^9^,^10^ However, a thorough history and physical can prove useful in placing SLE higher on the differential diagnosis list.

Our patient had the typical presentation of a case of pediatric SLE: fever, rash, fatigue, arthralgia or polyarthritis, anemia, thrombocytopenia, and renal disease.^10^ In adults, the presentation can be less severe and generally favors cardiopulmonary disease.^13^ Necrotizing vasculitis can occur in any tissue in SLE patients, including involvement of intestinal, cutaneous, pulmonary, and cardiac tissues.^14^ Lupus nephritis is common and may vary from mild renal insufficiency to frank renal failure.^15^ Deep vein thrombosis, pulmonary embolism, mesenteric ischemia, and cerebrovascular insults may occur in those with ***and*** without anti-phospholipid syndrome.^16^ A rare but important manifestation of SLE in both children and adult patients is shrinking lung syndrome, which is a progressive and irreversible restrictive lung disease that often presents with dyspnea and fatigue.

If SLE is suspected, laboratory studies that are both widely available and likely to result quickly during an ED visit include the basics: complete blood count, basic metabolic panel, chest radiograph, and urinalysis. Rheumatologic testing is less useful in the acute setting as these studies generally take hours to days to perform. ANA is considered sensitive but not specific, while anti-dsDNA and anti-Smith autoantibodies are considered confirmatory tests. If drug-induced lupus is suspected, then anti-histone autoantibody is the test of choice. In the setting of acute illness complicated by SLE, the emergency physician should address any acute medical conditions as usual. In addition to routine care, the clinician should consider consulting a rheumatologist and administering high-dose steroids for rapid immunosuppression, if an SLE flare is suspected.^17^

## FINAL DIAGNOSIS

Juvenile systemic lupus erythematosus.

## TAKE-HOME POINTS

- Patients with SLE:
  ○ More common in adult women, African Americans and Hispanics
- The diagnosis of systemic lupus erythematosus is made by the presence of four of the following:
  ○ Cutaneous involvement
  ○ Neurologic symptoms
  ○ Hematologic involvement
  ○ Renal impairment
  ○ Serositis
  ○ Arthritic symptoms
  ○ Positive immunological testing
- Immunological testing includes
  ○ ANA (sensitive but not specific)
  ○ Anti-dsDNA and anti-Smith (specific but not sensitive)
  ○ Anti-histone (in drug-induced SLE)
- Patients with SLE often have a disordered coagulation cascade.
  ○ 40–50% of patients with SLE have anti-phospholipid syndrome.
    ■ Deactivates prothrombin and proteins C and S
- In patients with acute manifestations of SLE, consider treating with high-dose steroids.

## Figures and Tables

**Table 1:** Hematology and coagulation studies of a 13-year-old girl with a fever.

Complete Blood Cell Count	
White blood cells	5.0 K/mcL
Hemoglobin	8.7 g/dL
Hematocrit	26.8%
Platelets	80 K/mcL

Coagulation Studies	

Prothrombin time	15.3 seconds
International normalized ratio	1.2
Activated partial thromboplastin time	39.2 seconds

**Table 2:** Chemistry results of a 13-year-old girl with a fever.

Serum Chemistries	
Sodium	139 mmol/L
Potassium	4.2 mmol/L
Chloride	104 mmol/L
Bicarbonate	29 mmol/L
Blood Urea Nitrogen	6 mg/dL
Creatinine	4.2 mmol/L
Magnesium	2.0 mg/dL
Phosphorous	4.0 mg/dL
Calcium	8.2 mmol/L
Total Protein	7.5 g/dL
Albumin	2.8 g/dL
Total bilirubin	0.4 mg/dL
AST	71 U/L
ALT	41 U/L
Alk Phos	92 units/L

Additional Labs	

LDH	1098 U/L
CRP	2.2 mg/L
ESR	125 mm/hour

*AST,* aspartate aminotransferase; *ALT*, alanine aminotransferase; *Alk Phos*, alkaline phosphatase; *LDH*, lactate dehydrogenase; *CRP*, C-reactive protein; *ESR*, erythrocyte sedimentation rate.
